# GROF: Indoor Localization Using a Multiple-Bandwidth General Regression Neural Network and Outlier Filter

**DOI:** 10.3390/s18113723

**Published:** 2018-11-01

**Authors:** Zhang Chen, Jinlong Wang

**Affiliations:** 1 College of Communications Engineering, PLA Army Engineering University, Nanjing 210007, China; wjl543@sina.com; 2 The 63th Institute, National University of Defense Technology, Nanjing 210007, China

**Keywords:** fingerprinting, general regression neural network (GRNN), indoor localization, K-nearest-neighbor (KNN), received signal strength (RSS)

## Abstract

In recent years, a variety of methods have been developed for indoor localization utilizing fingerprints of received signal strength (RSS) that are location dependent. Nevertheless, the RSS is sensitive to environmental variations, in that the resulting fluctuation severely degrades the localization accuracy. Furthermore, the fingerprints survey course is time-consuming and labor-intensive. Therefore, the lightweight fingerprint-based indoor positioning approach is preferred for practical applications. In this paper, a novel multiple-bandwidth generalized regression neural network (GRNN) with the outlier filter indoor positioning approach (GROF) is proposed. The GROF method is based on the GRNN, for which we adopt a new kind of multiple-bandwidth kernel architecture to achieve a more flexible regression performance than that of the traditional GRNN. In addition, an outlier filtering scheme adopting the k-nearest neighbor (KNN) method is embedded into the localization module so as to improve the localization robustness against environmental changes. We discuss the multiple-bandwidth spread value training process and the outlier filtering algorithm, and demonstrate the feasibility and performance of GROF through experiment data, using a Universal Software Radio Peripheral (USRP) platform. The experimental results indicate that the GROF method outperforms the positioning methods, based on the standard GRNN, KNN, or backpropagation neural network (BPNN), both in localization accuracy and robustness, without the extra training sample requirement.

## 1. Introduction

In the era of big data, growing commercial and industrial applications have generated a significant demand for location-based services (LBS). Accurate indoor location determination is an essential part of enabling extensive indoor location-based services (ILBS). Global navigation satellite systems (GNSS) as localization systems are satisfactory in outdoor scenarios, but are not reliable in indoor environments because of the degradation or absence of the satellite signal [1]. Hence, alternative indoor positioning systems (IPS) employing various technologies have been proposed, such as wireless local area network (WLAN) radio signals, Bluetooth signals, ultra-wide band (UWB), FM radio signals, radio-frequency identification (RFID), infrared, visual surveillance, ultrasound or sound, inertial measurement units (IMU), and magnetic fields [2].

These IPSs use different types of signal measurements such as time of flight (ToF) [3,4,5], time difference of arrival (TDoA) [4,5,6,7], angle of arrival (AoA) [8,9,10,11,12], channel state information (CSI), and received signal strength (RSS). For instance, Deepak Vasisht et al. [3] proposed an algorithm that can compute sub-nanosecond ToFs and locate with decimeter-level accuracy. The Active Bat system [6], based on the TDoA of ultrasound signals, can obtain accuracies within 9 cm for 95 percent of measurements. The SpotFi [8] system achieves a median accuracy of 40 cm by incorporating super-resolution algorithms that can precisely compute the AoA. The localization performances of the aforementioned state-of-the-art IPSs are remarkable and impressive. Nevertheless, the requirements of deploying special infrastructures (synchronized APs, acoustic badges, antenna arrays, etc.) limit the widespread application of these technologies. 

Conversely, the RSS-based IPSs are particularly popular for their inherent simplicity and pervasive support by most common wireless transceivers. The last decade observed a significant research effort directed towards indoor localization utilizing location fingerprinting techniques [13,14,15,16] that match the fingerprint of the RSS, which is location dependent. Fingerprinting is a kind of map-matching localization approach conducted in two phases. In an offline phase (survey), the fingerprint is the pattern of data trained from the RSS samples that are measured at pre-determined reference points (RP) labeled with their location information. All of the fingerprints of the target coverage make up a radio map that is an essential component of the fingerprint-based IPS. Secondly, once a radio map is constructed, the location of the target is determined using a positioning algorithm comparing the received real-time RSS with the stored fingerprints in the radio map. 

Some fingerprint-based IPSs employ a k-nearest neighbor (KNN) algorithm such as the RADAR [14] system, which is one of the landmark IPSs. Since then, many significant solutions [15] based on fingerprinting techniques keep coming forth, such as the Horus [16] system, which leverages the probabilistic model of the signal distribution and achieves better accuracy than that of the KNN methods at the expense of a heavy data payload. There are some systems employing support vector machine (SVM) algorithms to increase positioning accuracy [17], at the expense of high computing complexity. In the literature [18], a novel fingerprint fusing technique is proposed to improve the accuracy and robustness of localization, but the technique has tremendous data requirements and an intricate training process.

Furthermore, different kinds of artificial neural network (ANN) algorithms, including backpropagation neural networks (BPNNs) [19,20,21,22,23] and radial basis function (RBF) neural networks [24,25], have been applied to IPSs with notable progress. In recent years, with the upsurge of deep learning, deep neural network (DNN) algorithms have been employed in IPSs to increase the positioning accuracy and to reduce the generalization error [26]. Xuyu Wang et al. [27,28,29] proposed several deep learning schemes for CSI-based indoor fingerprinting applications. In the literature [30,31], the deep belief network (DBN) was exploited to reduce the influence of environment changes. However, DNNs require tremendous training samples to search the parameter space for optimal parameters, which is not feasible for IPSs, because of the time and computational resources’ costs.

The generalized regression neural network (GRNN) is a variant of the RBF neural network proposed in the literature [32]. The GRNN can achieve a high accuracy of both linear and nonlinear functional regressions based on the kernel estimation theory, which builds the necessary functional surface in a nonparametric fashion using the available data set [33]. Furthermore, the GRNN is easy to implement because of its much faster training procedure than that of other ANNs, such as BPNNs or DNNs. Moreover, GRNN exhibits a high robustness to sparse and noisy data. According to these advantages, the GRNN is attractive and has been widely applied in a variety of fields, including image processing, nonlinear adaptive controls, machinery fault diagnosis, and financial predictions [34,35,36].

To the best of our knowledge, there are only a few works using the GRNN in IPSs so far [37,38,39]. In the literature [37], the authors proposed an indoor localization algorithm using GRNN and weighted centroid localization with RSS data gathered at the access points from the reference nodes. The simulation and experimental results indicated that the proposed algorithm had good positioning performances in a line-of-sight (LOS) and non-line-of-sight (NLOS) condition. In the literature [38], the authors presented a RSS-based two-phase location estimation algorithm using GRNN with virtual grid points, which improved the localization accuracy compared with the localization accuracy obtained by only using the GRNN. In the literature [39], the GRNN was proved to be an effective method for the RSS-based wireless sensor networks (WSN) positioning system as well as a better convergence property than the traditional neural network. Nevertheless, the mentioned works did not pay enough attention to designing and optimizing the spreads’ value of the GRNN, which is essential to the performance of the GRNN. So, there is still potential for the GRNN to be exploited for IPS. Inspired by the widespread applications and the related works, in this paper, we introduced the GRNN into the fingerprint-based indoor localization scenario in order to obtain a high positioning accuracy, thereby avoiding tremendous training data or an exhaustive training process.

### 1.1. Problem Statement

Although the RSS-based localization methods are simple and easy to implement, the positioning accuracy and robustness performance are not satisfactory, as the RSS measurements are inherently sensitive to the dynamic environment. Moreover, indoor environments are rather complex and rich in multipaths [40,41], and have many interference factors such as pedestrian flow, layout rearrangement, and furniture displacement. Those interferences could make the measured RSS values fluctuate dramatically at the same location. Therefore, achieving excellent positioning accuracy and robustness is a problem for the RSS-based IPS.

There are two major ways to address this problem. As the radio map is essential to the fingerprint-based localization approach, area-covering and fine-grained RP placement is preferred to improve the localization accuracy. An intuitional approach is to design a comprehensive fingerprint database that contains information under different circumstances. Then, it can be combined with techniques such as DNNs, which can improve the accuracy and robustness of the positioning performance by leveraging a sufficiently large amount of training samples. Nevertheless, fingerprint surveying is a very difficult job, with intensive labor and time costs. Furthermore, a radio map should be calibrated frequently in order to adapt to environmental changes. This kind of method is not practical for pervasive applications, because of the expenses of tremendously increased surveying and processing overhead.

Another sophisticated way is to fuse the results of different kinds of fingerprints [42,43] or different fingerprint functions [44]. Although these kinds of localization frameworks benefit from promoting the positioning performance, the relatively complicated algorithm and the complex fingerprints database affect their practicability.

### 1.2. Contributions

In this paper, we propose a novel multiple-bandwidth generalized regression neural network with the outlier filter indoor positioning approach, named GROF, to improve the positioning accuracy and robustness.

Even though the fingerprints are commonly pre-located uniformly with equal intervals, the distances of the corresponding RSS vectors are multifarious. To adapt to the RSS distance distribution of the fingerprints, we developed a kind of multiple-bandwidth kernel training method to obtain multiple smooth values (denoted as spreads) for the pattern neurons. Compared to the standard GRNN, the proposed architecture can improve the fitting performance by dealing with dynamic data properties, without a heavy computational burden or complicated training workload.

In addition, we introduce an outlier filtering scheme by adopting the KNN method and the spatial correlation between fingerprints to enhance the localization robustness against the fluctuation of RSS measurements induced by the dynamic indoor environment. 

### 1.3. Organization

The remainder of the paper is organized as follows. Section 2 introduces the preliminary of our approach. Section 3 demonstrates the multiple-bandwidth spreads training procedure and analyzes the principle of the outlier filtering algorithm. In Section 4, we evaluate the performance of our approach through empirical experiments. Finally, concluding remarks are given in Section 5.

## 2. Preliminary

In this section, we present the preliminary of our approach. The fundamental of the GRNN algorithm is introduced first, and then the KNN algorithm is briefly discussed.

### 2.1. Generalized Regression Neural Networks

The general regression neural network (GRNN) developed by Specht in [32] is a kind of one-pass learning algorithm built on the notion of the kernel regression. Firstly, consider a nonlinear regression model defined by Equation (1).

(1) $$y_{i}=R\left(X_{i}\right)+\varepsilon \left(i\right),\;i=1,2,\ldots ,N$$

where *ε*(*i*) is an additive white-noise term of zero mean and variance *σ*^2^. The unknown function *R*(***X***) is the regression of *y* on ***X***, as shown by Equation (2).

(2) $$R\left(X\right)=E\left(y|X\right)=\frac{\int_{-\infty }^{\infty }yf\left(X,y\right)dy}{\int_{-\infty }^{\infty }f\left(X,y\right)dy}$$

where *f*(***X***, *y*) represents the known joint continuous probability density function of the random vector ***X*** and the variable *y*. However, in the actual scenario, the joint probability density function *f(**X***, *y)* is unavailable and only the training sample set {(***X****_i_*, *y_i_*)} (*i* = 1, 2, …, *N*) is available. Assuming that the observations *x*_1_, *x*_2_, …, *x_N_* are statistically independent and identically distributed (iid), the density estimate of *f(**X***, *y)* can be defined by using the nonparametric estimator, known as the Parzen–Rosenblatt density estimator, as shown by Equation (3).

(3) $$f\left(X,y\right)=\frac{1}{Nh^{m+1}}\overset{N}{\underset{i=1}{\sum }}k\left(\frac{x-x_{i}}{h}\right)k\left(\frac{y-y_{i}}{h}\right)$$

where $x\in {\mathbb{R}}^{m}\;\mathrm{and}\;y\in \mathbb{R}$, *k*(·) is a kernel, and the smoothing parameter *h* is a positive number that controls the size of the kernel. Using the property of the kernel *k*(·), we can obtain an estimation of the regression function *R*(*x*) from Equations (2) and (3).

(4) $$\hat{R}\left(X\right)=\frac{\sum_{i=1}^{N}y_{i}k\left(\frac{X-X_{i}}{h}\right)}{\sum_{j=1}^{N}k\left(\frac{X-X_{j}}{h}\right)}$$

The kernels are assumed to be hyperspherical in shape. The Gauss kernel function has better anti-noise ability and robustness, which is an attractive advantage to the RSS-based positioning algorithm.

When the kernel function is the multivariate Gaussian distribution, the regression estimator takes the form of Equation (5).

(5) $$\hat{R}\left(X\right)=\frac{\sum_{i=1}^{N}y_{i}\exp\left(-\frac{{\left|\left|X-X_{i}\right|\right|}^{2}}{2\sigma_{i}^{2}}\right)}{\sum_{j=1}^{N}\exp\left(-\frac{{\left|\left|X-X_{i}\right|\right|}^{2}}{2\sigma_{i}^{2}}\right)}$$

where *σ_i_* is called the smoothing parameter or spread, and it controls the width of the kernel. The estimated $\hat{R}\left(X\right)$ can be visualized as a weighted average of all of the observed values.

GRNN is a feedforward network that consists of an input layer, a pattern layer, a summation layer, and an output layer. Thus, the GRNN learns mapping from an input domain containing *X*, to an output codomain containing *Y*, where either space can be multidimensional. 

**Figure 1:**
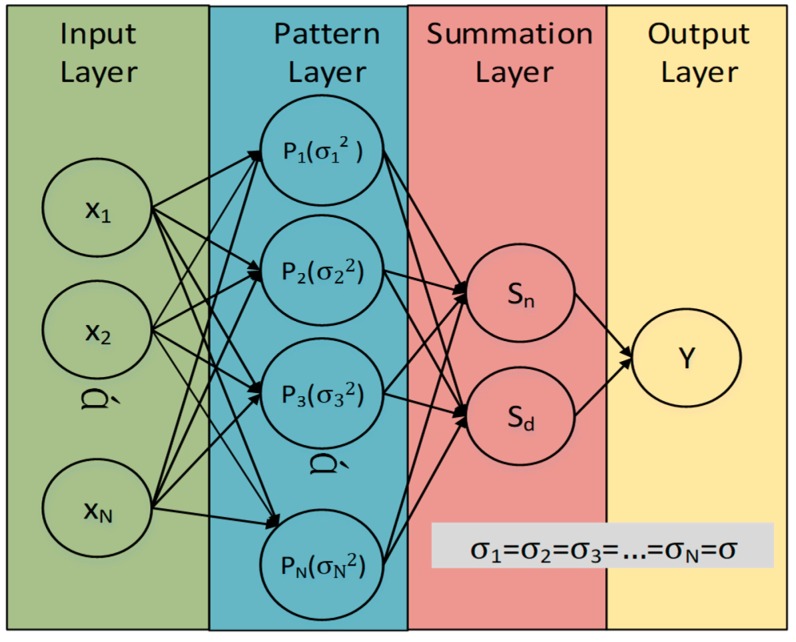
Standard generalized regression neural network (GRNN) block diagram.

In our indoor localization scenario, the structure of the GRNN is depicted in Figure 1. The GRNN can be regarded as an incremental learning system that simplifies the RSS-based localization by providing a way to learn the intricate relationship between the measured signal vector and position. 

In the input layer, there are N neurons that are fed with the input vector ***X*** = [*x*_1_, *x*_2_, …, *x_N_*]*^T^* and transmitted to the pattern layer directly.

In the pattern layer, the quantity of neurons is exactly equal to the pattern number of the input training sample. Each neuron in this layer can be regarded as an individual Gauss kernel, as defined by Equation (6).

(6) $$P_{i}=\frac{1}{\sqrt{2\pi }\sigma }\exp\left[-\frac{{\left(X-X_{i}\right)}^{T}\left(X-X_{i}\right)}{2\sigma^{2}}\right]\;$$

where ***X****_i_* is the subspace central point of *i*-th kernel. In the standard GRNN, all of the pattern neurons are assumed to share the same spread value *σ*.

The summation layer consists of two kinds of neurons, as shown by Equation (7).

(7) $$\mathrm{neuron}\;\mathrm{pair}=\{\begin{array}{c} S_{n}=\overset{N}{\underset{i=1}{\sum }}y_{i}p_{i}\\ S_{d}=\overset{N}{\underset{i=1}{\sum }}p_{i} \end{array}$$

where *y_i_* denotes the label value of the *i*-th kernel.

Finally, the estimation value is calculated, using Equation (5), at the output neurons.

### 2.2. K-Nearest-Neighbor Algorithm

The KNN algorithm is among the simplest of all machine learning algorithms, and it plays a part in RADAR and many other localization systems. The algorithm is easy to implement by comparing the similarity metric (such as the Euclidean distance) between the online data and the prebuilt database, according to the least square criterion, to find the *k* nearest fingerprints. Then, these *k* candidates are averaged, and the distances are adopted as weights. In the positioning application, the KNN algorithm is processed in following steps:

Calculate the Euclidean distance, *D_i_*, between the measured signal strength, *rss_i_*, and the stored fingerprints, *RF_i_*, as shown by Equation (8). Select *k* fingerprints that have the smallest distance to the real-time RSS.

(8) $$D_{i}=\overset{n}{\underset{i=1}{\sum }}\left|\left|rss_{i}-RF_{i}\right|\right|$$

Estimate the target location by using Equation (9).

(9) $$\hat{C}=\frac{\sum_{i=1}^{k}\frac{1}{D_{i}}C_{i}}{\sum_{i=1}^{k}\frac{1}{D_{i}}}$$

where *C_i_* is the location corresponding to the selected fingerprint.

The KNN algorithm is effective and easy to be realized for IPSs. Nevertheless, the KNN algorithm is sensitive to the fingerprints and the choice of the parameter *k*, which affects the positioning accuracy significantly [14].

## 3. Rationale and Methodology

In this section, we present the rationale and methodology of the proposed approach. We first analyze the existing challenges of the standard GRNN algorithm in IPSs. Then, we will show the framework of the GROF method, introduce the multiple-bandwidth kernel spread training process, and discuss the outlier filter algorithm.

### 3.1. Challenge

The spread value *σ* of the pattern neuron in Equation (5) controls the smoothness of the regression surface and is essential to the performance of the GRNN. If the spread values are too small, the regression surfaces become very irregular and spiky, and resemble the nearest neighbor regression. Meanwhile, the large values result in over smoothed surfaces that are rather similar to linear fitting. In a standard GRNN architecture, every variable in the Gaussian kernel of all of the pattern neurons is supposed to share the same spread value with the others, so that there is only one parameter for training. Although this assumption is beneficial for simplifying the training procedure, it is not rational in some actual occasions, especially in complex indoor environments. To achieve a more flexible adaptation of the regression surface, the configurations of the kernels should be multiple-bandwidth. Some previous works [45] use clustering algorithms to train multiple-bandwidth spread values. However, it is very challenging to select the bandwidth size and optimize the corresponding spread value for the indoor localization scenario. 

In complex indoor environments, there are many interferences, including (but not limited to) the multipath effect, the shadowing effect, and noise. Those interferences give rise to troublesome, dramatic fluctuations of RSS values. In short, the trained network cannot precisely match the input data with the fingerprints dataset anymore. In this case, the degradation and instability of the positioning accuracy is inevitable. Another large challenge is to promote the resistibility of the environmental interferences of the GRNN algorithm.

### 3.2. Framework of GROF Method

**Figure 2:**
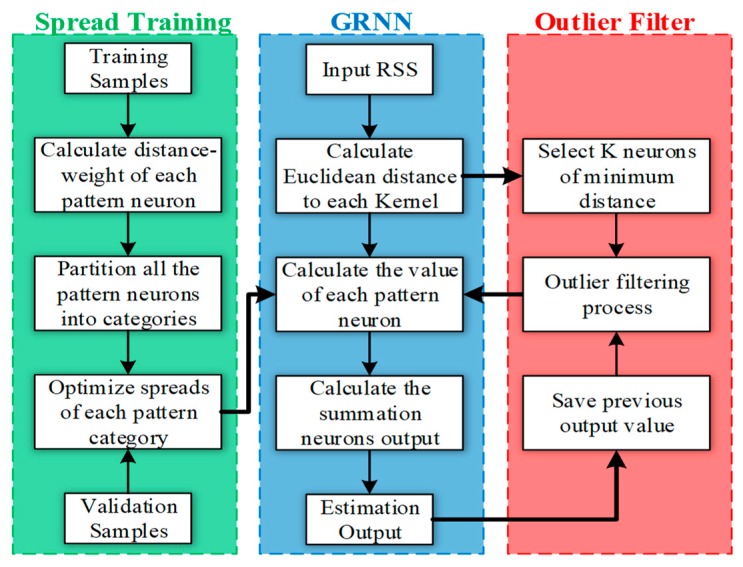
GROF method flow chart.

For the above reasons, our purpose is to present a lightweight fingerprint algorithm that is sufficiently precise and robust. The flow diagram of the proposed GROF positioning framework is depicted by Figure 2.

There are three main blocks in this localization framework: the spread training block, the GRNN block, and the outlier filter block.

**Figure 3:**
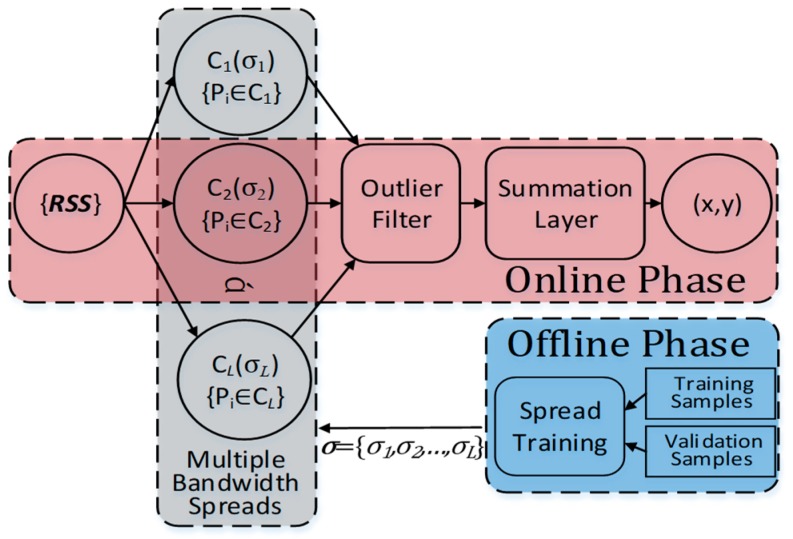
Outlier filter indoor positioning approach (GROF) block diagram.

In our indoor localization scenario, the proposed GROF structure is shown by Figure 3. There are four input neurons injected with measured RSS values. In the spread training block, the pattern neurons are partitioned into different categories, which own spread values different from others, according to the RSS vectors and the locational relationship of training fingerprint samples. The category number *L* and the multiple bandwidth spread values {*σ_i_*|*i* ∈ [1, *L*]} are obtained according to the procedure explained in Section 3.3.

During the online phase, the measured RSS vectors {RSS} are fed into the trained network. The output values of all the pattern neurons can be obtained by (15). Before flowing to the summation layer, the data set {*p_i_*} is refined by the outlier filter block described in Section 3.4.

### 3.3. Multiple-Bandwidth Kernel Spread Training

In this section, we present a kind of heuristic method to train the spread values of the multiple-bandwidth kernel network.

As the GRNN estimation is derived from the Parzen–Rosenblatt density estimation, we can formulate the multivariate Gaussian kernel using Equation (10).

(10) $$P\left(x\right)=\frac{1}{n}\overset{n}{\underset{i=1}{\sum }}\frac{1}{{\left(\left|\Sigma_{i}\right|\sqrt{2\pi }\right)}^{k}}\exp\left(-\frac{1}{2}{\left(x-x_{i}\right)}^{T}\Sigma_{i}\left(x-x_{i}\right)\right)$$

Given a collection of fingerprints, *F* = [*f*_1_, *f*_2_, …, *f_n_*] ∈ ℝ*^k^*^×*n*^, the corresponding kernel quantity is *n* and there are *k* variables in each kernel. The bandwidths of the kernels are controlled by the diagonal matrix, $\Sigma_{i}=\mathrm{diag}\left\{h_{1},h_{2},\ldots ,h_{k}\right\}$, which contains the spread values, *h*, of each individual variable in kernel *i*. During the spread design process, the major challenge is how to compromise the fine-grained spread design and the training workload alleviation.

The multiple-bandwidth spread training method is implemented in following steps:

**Step 1:** Calculate the distances of the RSS vectors between different pattern neurons, in order to find the adjacent kernels.

(11) $$d_{ij}=\left|\left|rss_{i}-rss_{j}\right|\right|\;i\neq j\;1<i,\;j\leq n$$

According to the traditional calculation approach, we have to calculate the RSS vector distances from every pattern neuron to the others, thus obtaining *n*(*n* − 1)/2 different distance values. The calculation complexity increases geometrically with the growth of the pattern number. To simplify this calculation procedure, we hold the assumption that the adjacent kernels are supposed to be the fingerprints that are neighbors in the ground truth. As in the line-of-sight (LOS) condition, it makes sense that the fingerprints are more similar when they are geographically closer. According to this assumption, the necessary calculation amount is significantly reduced and alleviates the computational overhead. As RPs are distributed in a rectangle area in most cases, the proposed algorithm can reduce the calculation amount by at least $\left(n+\sqrt{n}\right)/4$, referring to Appendix A. 

**Figure 4:**
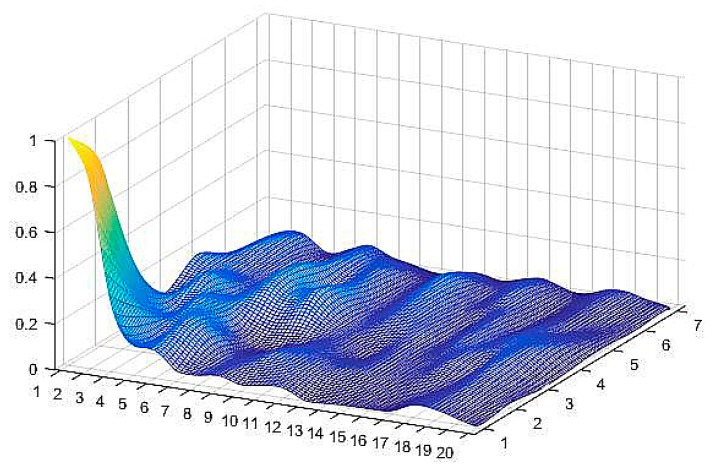
Pattern neurons’ distance–weights distribution diagram (distance–weights are regularized by the max–min method, labels on the *x* axis and *y* axis are the indices of reference points (RPs)).

**Step 2:** We partition the pattern neurons into categories by referring to the distance–weights distribution. The distance–weight of the *i*-th pattern neuron is defined according to Equation (12).

(12) $$w_{i}=\;\frac{1}{k}\overset{k}{\underset{j=1}{\sum }}d_{ij}$$

where *k* stands for the number of neighbors. Once the distance–weights set, *W* = [*w*_1_, *w*_2_, …, *w_n_*] ∈ ℝ^1*×n*^, has been determined, the distributional diagram of all of the patterns’ distance–weights is available. There are 140 pattern neurons on the basis of fingerprints that are located in the *x–y* plane. The distribution surface is generated by the set *W* with the triangulation-based natural neighbor interpolation method, as shown in Figure 4. The data was from our training sample data set. The distance–weights are calculated by Equation (12), and are then regularized by the max–min method for convenience. It is very intuitional that the distance–weights of the training data are distributed on a rough surface with a steep crest in the bottom left corner of it. The peak indicates where the maximum distance–weight pattern neuron is.

Instead of utilizing a complex algorithm such as clutering, we determined the category quantity *C* according to Equation (13).

(13) $$C=\mathrm{INT}\left(\frac{|\left|W\right|{|}_{\infty }}{\beta \times \mu_{W}}\right)\;s.t.\;0<\beta \times \mu_{W}\leq |\left|W\right|{|}_{\infty }$$

where $|\left|W\right|{|}_{\infty }$ is the maximum distance–weight in set *W* and *μ_w_* is the mean. As the distance–weights data are regularized by the max–min method, we can obtain $|\left|W\right|{|}_{\infty }=1$. The interval of one category is given as Δ = *β × μ*. The parameter *β* is an intermediate variable introduced to control the quantity of spreads. As *μ* is the normalized mean value of distance–weight calculated by Equation (12), *β* can be considered as a zoom factor to ensure that the value of Δ is within a reasonable range. The kernels in each interval form one category and share the same spread value. It is possible that some categories are null and no kernel falls into those intervals. Thus, the actual number of spread categories is likely to be less than *C*.

**Figure 5:**
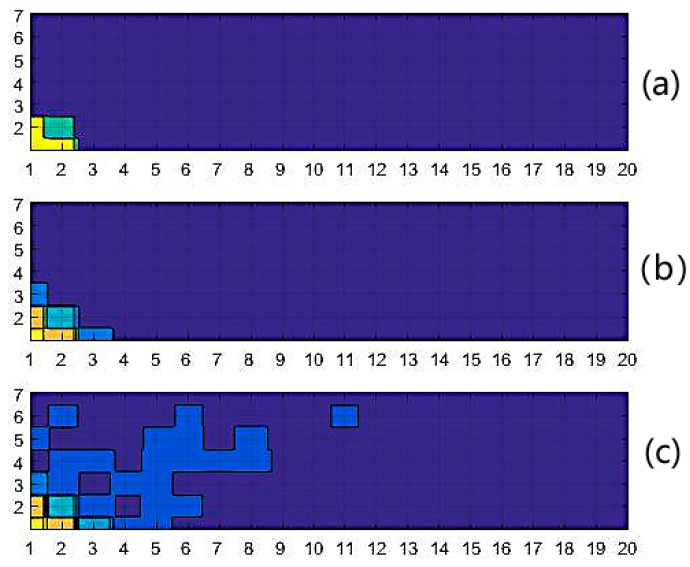
Pattern neurons partitioned with different steps (labels on the *x* axis and *y* axis are indices of RPs). (**a**) Existing thre categories when Δ = 0.4; (**b**) existing five categories when Δ = 0.2; (**c**) existing six categories when Δ = 0.1.

The spread quantity and the corresponding distribution are very flexible by tuning Δ, according to the requirement of the fitting performance. Figure 5 depicts the dynamic partition of the given fingerprint set under different Δ*_s_*, where different categories are distinguished by colors. The distribution of the color blocks is in accordance with our hypothesis that adjacent fingerprints have similar distance–weights.

**Step 3:** Obtain the optimal spreads by applying a gradient-based optimization scheme.

Our iterative algorithm is based on the following assumptions:

(1) The spread value of kernel *i* should be proportional to the mean of {*w*}*_i_*.
(2) The categories with larger sizes are more significant to the GRNN performance.
(3) The spread diagonal matrix can be extended from the category spread, according to the distance–weights scaling relationship of each variable in the kernel.

Assuming that the kernels of the training samples are partitioned into *l* categories, the new sequence of categories is arranged according to the descending order of their distance–weights, which is denoted as {*c*_1_, …, *c_l_*| *l* ≤ *C*}.

The initial spread value of each category is defined as follows:

(14) $$\sigma_{i}^{\left(0\right)}={\left(\gamma_{i}\times \frac{a_{i}}{b_{i}}\right)}^{1/2},\;\{\begin{array}{c} a_{i}=|\left|c_{i}\right|{|}_{1}\\ b_{i}=|\left|c_{i}\right|{|}_{0} \end{array},\;i\in \left[1,l\right]$$

where *c_l_* is the distance–weight set of the *i*-th category, *a_i_* is the distance–weight sum of the *i*-th category, *b_i_* is the member quantity of the *i*-th category, and the initial value of *γ_i_* is set to 0.5. We can modify Equation (5) as follows:

(15) $$\hat{y}(X)=\frac{\sum_{j=1}^{l}\sum_{i=1}^{b_{i}}\frac{y_{j}}{\left|\sigma_{j}\right|}\exp(-\frac{{\left(X-X_{i}\right)}^{T}\Sigma_{i}(X-X_{i})}{2\sigma_{j}^{2}})}{\sum_{j=1}^{l}\sum_{i=1}^{b_{i}}\frac{1}{\left|\sigma_{j}\right|}\exp(-\frac{{\left(X-X_{i}\right)}^{T}\Sigma_{i}(X-X_{i})}{2\sigma_{j}^{2}})}\;$$

where ***X*** stands for the input vector data of the training sample set, and ***X****_i_* is the center value of the *i*-th kernel. Σ*_j_* is the diagonal matrix of kernel *i* in category *j*. The initial values of Σ*_j_* are determined by referring to the average spread value of each variable in the kernel.

Assuming that the initial spread of set {*c_v_*} is *σ_v_*, kernel *i* belongs to set {*c_v_*}. The distance–weight of every variable in kernel *i* can be decomposed from Equation (11). The average distance–weight of each dimension in set {*c_v_*} is normalized. The diagonal matrix is obtained based on the weighted average method following Equations (16)–(19).

(16) $$d_{ij,q}=\left|\left|rss_{i,q}-rss_{j,q}\right|\right|\;i\neq j\;q\in \left[1,p\right]\;$$

(17) $$w_{i,q}=\;\frac{1}{k}\overset{k}{\underset{j=1}{\sum }}d_{ij,q}\;q\in \left[1,p\right]\;$$

(18) $$w_{q}=\;\frac{1}{b_{v}}\overset{b_{v}}{\underset{i=1}{\sum }}w_{i,q}\;q\in \left[1,p\right]\;$$

(19) $$\Sigma_{i}=\frac{\sigma_{v}}{\sum_{q=1}^{p}{\left(w_{q}\right)}^{2}}\left[\begin{array}{ccc} {\left(w_{1}\right)}^{2} & \cdots & 0\\ \vdots & \ddots & \vdots \\ 0 & \cdots & {\left(w_{p}\right)}^{2} \end{array}\right]$$

The total estimation error of the m-length training samples is defined as follows:

(20) $$E=\overset{m}{\underset{t=1}{\sum }}\frac{1}{2}{\left(\hat{y_{t}}-{\bar{y}}_{t}\right)}^{2}$$

where $\hat{y_{t}}$ is the estimation value and ${\bar{y}}_{t}$ is the corresponding target value. We calculated the gradient of the estimation error by differentiating with respect to the current spread *σ_i_*, as in Equation (17).

(21) $$\frac{\partial E}{\partial \sigma_{i}}=\overset{m}{\underset{t=1}{\sum }}\left(\hat{y_{t}}-{\bar{y}}_{t}\right)\frac{\partial \hat{y_{t}}}{\partial \sigma_{i}}\;\;i\in \left[1,l\right]$$

The traditional gradient descent algorithm is not the emphasis of this paper, and some details have been described in a similar scheme [42]. 

Unlike other methods, the optimal spread of each category in our method is attained one by one, similar to completing a jigsaw puzzle. The highlight appears in the training and validation phase. Conventionally, the optimal spreads are supposed to minimize the target function, Equation (20), with the whole validation data set. In our approach, the target function is dynamic for different spreads, as their validation data sets are selected specially. We also partition the validation data by referring to the principle expounded in step 1.

(22) $$E\left(c_{i}\right)=\overset{b_{i}}{\underset{t=1}{\sum }}\frac{1}{2}{\left(\hat{y_{t}}-{\bar{y}}_{t}\right)}^{2},\;i\in \left[1,l\right]$$

Firstly, we selected the spread value of {*c*_1_} as the benchmark. When the iteration begins, the spread of *σ*_1_ is updated with the gradient descent algorithm, as in Equation (23), while the other spread values are varying in proportion to *σ*_1_, according to Equation (24).

(23) $$\sigma_{i}^{\left(t+1\right)}=\sigma_{i}^{\left(t\right)}-\varepsilon \frac{\partial E_{i}}{\partial \sigma_{i}}\;$$

(24) $$\sigma_{j}^{\left(t+1\right)}=(\frac{\sigma_{i}^{\left(0\right)}}{\sigma_{j}^{\left(0\right)}})\sigma_{i}^{\left(t+1\right)},\;j>i$$

where *ε* is the step coefficient that controls the fitting accuracy and convergence speed of the iterative algorithm. In particular, the validation data set that is injected into the target function has a similar distance–weight to set {*c*_1_}. The optimal spread value $\hat{\sigma_{i}}$ is calculated according to Equation (25).

(25) $$\hat{\sigma_{i}}=\arg\underset{i\in \left[1,l\right]}{\min}E\left(c_{i}\right)$$

Once the iteration is done, *σ*_1_ will be treated as a constant, and the validation data that is similar to {*c*_2_} would be supplemented to the target function. Repeat the above process until all of the spreads are acquired.

In the NLOS cases, as some adjacent fingerprints may be separated by walls, doors, pillars, or some other obstacles, their RSS vectors may be significantly different from each other. Thus, the assumption that the fingerprints are more similar when they are geographically closer is no longer valid.

**Figure 6:**
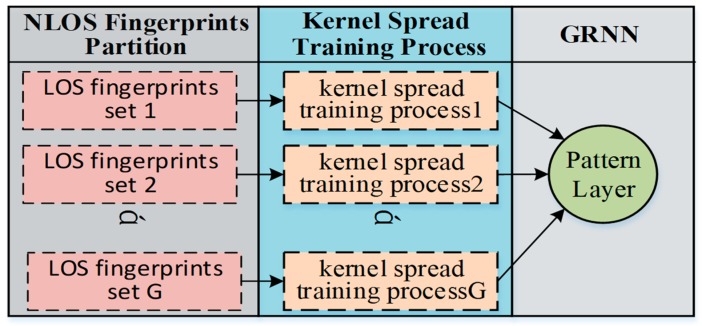
Fingerprints processing diagram in the non-line-of-sight (NLOS) case.

Fortunately, the pattern neuron of the GRNN is independent from the others, and the spread value of each pattern neuron can be calculated individually. Therefore, we can partition the NLOS object area into several subareas. The partition principle is to guarantee that each subarea is convex so that each RP in it is in LOS to each other, as shown in Figure 6. To avoid confusion, here, the mentioned LOS actually means that there is no obstacle between each RP and its neighbor RPs rather than APs. The convex contour of the subarea guarantees that most of the RPs have sufficient LOS neighbors. In this way, the geographical correlation of the fingerprints, that the adjacent kernels are supposed to be neighbors in the ground truth still works in each subarea. Hence, the multiple-bandwidth kernel spread training process can be carried out in the subareas, as in the LOS case. 

The proposed NLOS area division method is simple and straightforward. The object area can be partitioned into several subareas according to the actual layout. As long as it is gathering all of the trained pattern neurons from all of the subareas, the spread training procedure in NLOS case is successfully achieved.

In conclusion, the proposed multiple-bandwidth kernel spread training method leverages the geographical correlation of the fingerprints, that the adjacent kernels are supposed to be neighbors in the ground truth. It is flexible and significant that the tunable spread scale is beneficial to achieve a good tradeoff between the performance and complexity for the positioning task. Furthermore, when the object area is very large or in the NLOS condition, the proposed method is still working, by dividing the area into several small and fingerprints-convex areas. Then, the spread training process runs in each subarea separately, and generates a part of the neurons in the pattern layer of the GRNN. The proposed algorithm does not need the information of the APs’ precise positions, it just needs the layout of the target area, which is usually a prerequisite for fingerprint-based IPSs.

### 3.4. Outlier Filter Algorithm

In this section, we present the detailed procedure of the outlier filter algorithm.

The first step is to find the nearest RPs to the target. No matter whether the GRNN or KNN algorithm is used, the pattern whose value is similar to the input has more effect on the result estimation, and they are usually considered to be the nearest neighbors. Therefore, it is very important to recognize the nearest fingerprints for the indoor localization scenario.

Theoretically, the reference point whose fingerprint has the shortest distance to the input RSS value is supposed to be the nearest neighbor. However, it is not exactly in the temporal dynamic indoor environment, where human presence and mobility interfere with the RSS measurement dramatically. According to experience and the experimental results, the positioning accuracy would deteriorate when the calculated nearest neighbor is false. To address this problem, we propose an outlier filtering scheme to identify whether these candidate patterns are real neighbors of the target location.

**Figure 7:**
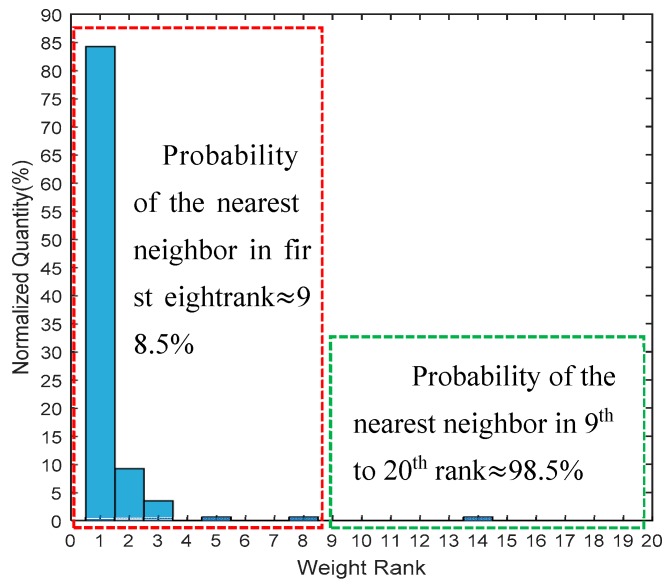
Weight rank evaluation of real nearest pattern.

The Euclidean distance between the RSS vector of the input data, *rss_in_*, and the RSS vector of all of the fingerprint data can be calculated by using Equation (11). Select *k* fingerprints from the set {*f_i_*}, and ∀*I* ∈ [1, *n*] as the neighbor candidates corresponding to the *k* minimum distance. The candidate quantity *k* is predefined as similar to in the KNN algorithm, and the main principle is to guarantee that the nearest neighbor to the target location is among the reference nodes corresponding to these *k* minimum *p_i_*. Although there is no analytical solution for the optimal value of *k*, it can be determined experimentally for a given condition. In order to obtain a convincing result, we evaluated the distance rank of the real nearest neighbor in set {*f_i_*} with the given data set, which is composed of 9100 individual sample data collected under multi-conditions. The result is shown in Figure 7, which depicts that the distance rank of the real nearest neighbor fingerprint was within 8 in 98.5% of the occasions, while being in first place 83% of the time. Referring to this analysis result, we define *k* as 8 in the following discussion.

The next step is to sort the *k* candidate fingerprints in ascending order, based on the distance rank, which is denoted as *F_c_* = [*c*_1_, *c*_2_, …, *c_k_*]*^T^*, and list the corresponding location coordinates {(*x_i_*, *y_i_*)}(*i* ∈ [1, *k*]). The spatial distances between these patterns are easily obtained and expressed in matrix *V*. Thus,

(26) $$V=\left[\begin{array}{ccc} v_{11} & \cdots & v_{1k}\\ \vdots & \ddots & \vdots \\ v_{k1} & \cdots & v_{kk} \end{array}\right]\;$$

where

(27) $$v_{ij}=\;\sqrt{{\left(x_{i}-x_{j}\right)}^{2}+{\left(y_{i}-y_{j}\right)}^{2}}\;i,j\in \left[1,k\right]\;$$

We can learn from Figure 7 that the first candidate, *c*_1_, is most likely to be the real nearest fingerprint, while *c*_2_ has a one-in-ten chance. Different processing strategies are conducted according to the spatial relationship between *c*_1_ and *c*_2_. Given parameter *v_th_*, which is defined as the adjacency threshold, it represents the maximum distance of the credible candidates to *c*_1_. The optimal threshold value, *v_th_*, can be determined by using a cross-validation method, such as the leave-one-out (LOO) method.

If *v*_12_ ≤ *v_th_*, then *c*_2_ is supposed to be adjacent to *c*_1_. In this situation, even though *c*_2_ was the nearest one, the resulting error is tolerable. Otherwise, *c*_1_ and *c*_2_ are far apart in the ground truth, which would result in an unacceptable positioning error. We adopted a more cautious approach to identify the nearest neighbor between *c*_1_ and *c*_2_. First, we calculated the square of the Euclidean distance between the RSS vector of the input data *rss_in_* and the RSS vector of *c*_1_ and *c*_2_, such that

(28) $$|\left|rss_{in}-rss_{c}\right|{|}_{2}^{2}=\overset{p}{\underset{q=1}{\sum }}{\left(rss_{in,q}-rss_{c,q}\right)}^{2}=\overset{p}{\underset{q=1}{\sum }}d_{c,q}\;$$

A score scheme is addressed by comparing each dimension of the RSS value according to

(29) $$s_{q}=\{\begin{array}{ll} 1, & \mathrm{if}\;d_{c1,q}\leq d_{c2,q}\\ 0, & \mathrm{otherwise} \end{array},\;\forall q\in \left[1,p\right]$$

When the *q*-th distance component of *c*_1_ is not greater than that of *c*_2_, score one, and the total score ranges from 1 to *p*. Furthermore, we consider the output location of the last estimation as another constraint. As the target’s movement velocity is limited, the upper limit of the distance between two successive estimation outputs is denoted as the vigilance parameter, *ρ*. We define the constraint function *ξ* as follows:

(30) $$\xi =\{\begin{array}{ll} 0, & v_{20}>\rho \\ 1, & \mathrm{otherwise} \end{array}$$

where *v*_20_ denotes the distance between estimation of *c*_2_ and the estimated result at the previous time.

The final score is as follows:

(31) $$S=\overset{p}{\underset{q=1}{\sum }}s_{q}-\xi$$

If *S* = 0, we assume that *c*_2_ is more likely to be closer to the target, and we exclude *c*_1_ from the candidate set. Otherwise, *c*_1_ defends its nearest neighbor rank. The determined nearest neighbor fingerprint is set as the benchmark in order to identify the outliers in *F_c_*, by referring to the updated matrix, *V*, and the adjacency threshold, *v_th_*, such that

(32) $$z_{i}=\{\begin{array}{ll} ‘Ture’, & v_{1i}\leq v_{th}\\ ‘Fake’, & \mathrm{otherwise} \end{array}\;,\forall i\in \left[2,k\right]$$

We listed the “fake” candidate fingerprint index in the outlier set *Z*, which is sent to the GRNN block, and the corresponding neuron is excluded from the summation layer.

**Figure 8:**
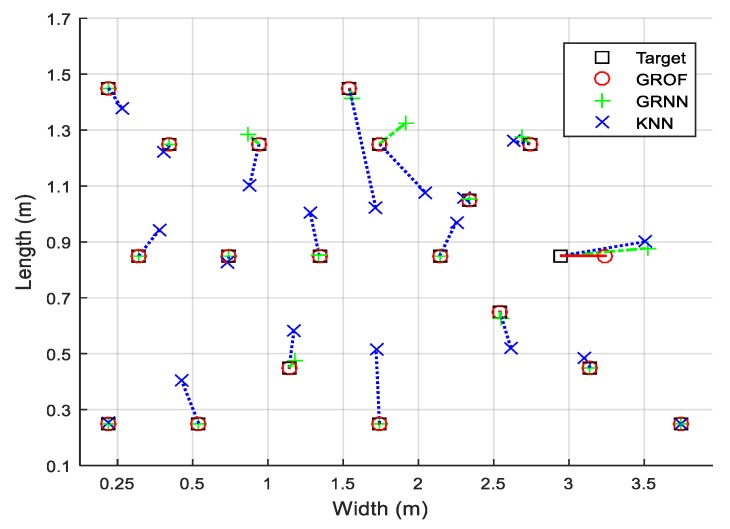
Localization estimation deviation comparison of twenty test points between different algorithms.

As shown in Figure 8, we randomly select 20 RPs to compare the localization performances. The combination of the GRNN and the outlier filtering mechanism is named GROF, while the combination of the KNN and the outlier filtering mechanism is named KOF. With the help of the outlier filter algorithm, both the GRNN and the KNN method achieve better localization accuracy and enhance the system’s robustness against RSS fluctuations. The localization results indicate that the GROF method significantly alleviates the deviation of the estimation results compared with the KOF, GRNN, and KNN methods.

## 4. Experimental Results and Discussion

In this section, we introduce the details of the experimental implementation of GROF. The RSS samples were collected by a Universal Software Radio Peripheral (USRP) platform, so as to obtain a fine-grained measurement for tracking the variation of the signal [43]. We compare the localization performance of GROF with the KNN, GRNN, and BPNN algorithms.

### 4.1. Experimental Environment and Implementation

We built the testbed with several USRP-2920s of NIs in a typical laboratory environment. One USRP is used for transmitting the signal through the antenna fixed on a remote-controlled robot, which is moving within the target area, while the other USRPs are in charge of handling the signal that is received by the monitoring antenna. We deployed both the transmitting antenna and the monitoring antennas at the same height, about 1 m above the ground. The software of the positioning algorithm was developed with the C++ application programming interface.

**Figure 9:**
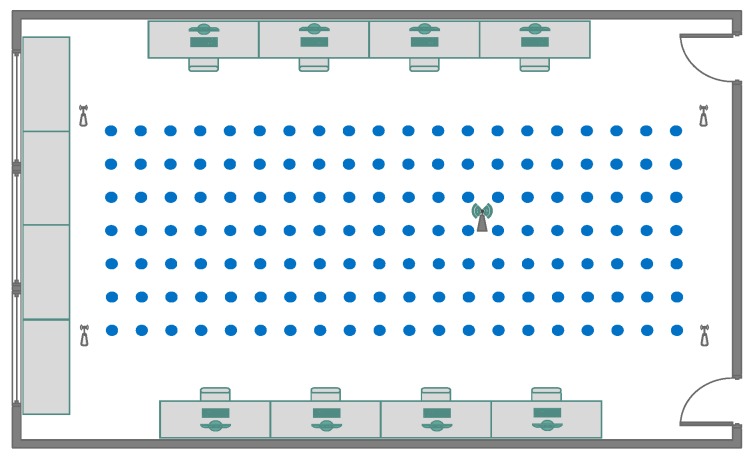
The Testbed.

The experiments were conducted in office 215, located on the second floor of the Electronic and Information Engineering Building on the campus of Nanjing University of Aeronautics and Astronautics. As shown in Figure 9, we deployed four monitoring antennae in the corners of a rectangular platform with the size of 3 × 5 m^2^. A 0.2 m spacing grid is defined over this two-dimensional area, and reference points are placed at the crossings of each gird. The signal frequency of the transmitter was set to 2.01 GHz, with 1 MHz modulated bandwidth in the Quadrature Phase Shift Keying (QPSK) modulation mode. We did not choose any standardized technology like Wi-Fi or LTE signal, so as to avoid interference in the testing signal generated by USRP.

**Figure 10:**
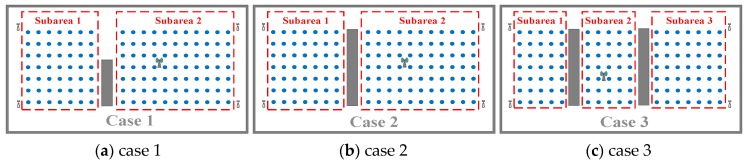
The testbed in NLOS condition.

For the NLOS discussion, in order to be without a loss of generality, we rearranged the experimental deployment in three different cases in order to contain more indoor environment situations. As shown in Figure 10a, the first case is to simulate the situation that there are some obstacles like pillars or short walls existing in the object area, and a part of the monitoring antenna remains as LOS. The second case is to simulate the situation that the object area is consisted with rooms separated by a wall, and the monitoring antennas are deployed in the rooms, as shown in Figure 10b. The third case is to simulate the situation that the object area is consisted with rooms separated by a corridor, and the monitoring antenna (APs) are only deployed in the rooms, as shown in Figure 10c. There is about 12 dB attenuation of RSS induced by the obstacle around 2 GHz, corresponding to the 0.3 m thick brick walls [46].

Ut supra, we used multiple receivers to obtain a signal from the object source, and all the received data were gathered to the processing program on a computer. Our testbed is similar to the WSN in the literature [38]. In another related work [37], the experimental testbeds were different, where there is one receiving node and multiple transmission sources (e.g., Access points). However, the data structures of the fingerprints, which were composed of vectors of received signal strength from their experiments, are similar to those in the WSN case. No matter what kind of signal it is, it could be processed by our proposed approach successfully.

### 4.2. Survey Phase

When the experiment preparation was done, the following survey task was implemented to gather the fingerprint data and store it in the format of *f*(*i*) = {*rss*1(*i*), *rsss*2(*i*), *rss*3(*i*), *rss*4(*i*), *X*(*i*), *Y*(*i*)}. Each sample contains an RSS vector with the input and coordinate values of the corresponding reference node as the target for the GRNN. We collected the fingerprint data while a robot equipped with the omnidirectional transmitting antenna passed through every RP. One whole fingerprints set contains 140 *f*(*i*) in our experiment.

During the survey phase of the LOS case, as shown in Figure 9, the RSS data were measured over five days in a dynamic indoor environment with random people motion. For every RP, over 100 snapshots of the signal RSS samples were collected at different times. From this, 80 fingerprint sets constituted the training data set. Another 10 fingerprint sets collected on a different day than the training data were used for the validation and tuning during training phase. Finally, another 10 fingerprint sets that were collected separately from the training and validation days were used to evaluate the localization accuracy.

During the survey phase for the NLOS condition, as we rearranged the experimental testbed in three different cases by deploying obstacles in the RP area as shown in Figure 10, the RSS data were measured respectively in dynamic indoor environments. For every RP, over 24 snapshots of signal RSS samples were collected. From this, 16 fingerprint sets constituted the training data set. Another four fingerprint sets collected on a different day than the training data were used for validation and tuning during training phase. Finally, another 4 fingerprint sets that were collected separate from the training and validation days were used to evaluate the localization accuracy.

### 4.3. Training Phase 

In this subsection, we first present the training algorithm and then evaluate the respective influences of different fingerprint set scales, preprocessing methods, and spread value optimizations on the positioning accuracy.

#### 4.3.1. Spread Optimization Algorithm

The training procedure of the GROF is illustrated as Algorithm 1. The goal of training is to obtain appropriate spread values, and the details have been expounded in Section 3.3.

**Algorithm 1.** Spread Optimization 
**Input:**
*n*: number of fingerprint nodes;
*p*: number of input variables;
***F*** = [*f*_1_, *f*_2_, …, *f_n_*] ∈ R*^k×n^*: the training fingerprint set;
***RSS_i_*** = [*rss_i_*_,1_, *rss_i_*_,2_, …, *rss_i_*_,*p*_]: the RSS collections of *i*-th fingerprint.

**Output:**

*l*: category quantity of spread values;
***H*** = [*σ*_1_, *σ*_2_, *σ_l_*]: the spread set of every category.
**Σ** = [Σ_1_, Σ_2_, …, Σ*_l_*]: the diagonal matrix set of every category.
1: **for** the *i*-th kernel, *i* = 1, …, *n*
**do**
2: Calculate distances of RSS vector between *i*-th kernel and its neighbors.

$d_{ij}=\left|\left|rss_{i}-rss_{j}\right|\right|\;i\neq j\;1<i,\;j\leq n$

3: Calculate distance-weight of *i*-th kernel.

$w_{i}=\;\frac{1}{k}\overset{k}{\underset{j=1}{\sum }}d_{ij}$

4: **end for**
5: Partition pattern neurons into ***C*** categories referring to distance-weights distribution.

$C=\mathrm{INT}\left(\frac{|\left|W\right|{|}_{\infty }}{\beta \times \mu_{W}}\right)\;s.t.\;0<\beta \times \mu_{W}\leq |\left|W\right|{|}_{\infty }$

6: Rearrange categories sequence according to the descending order of their distance-weights: {*c*_1_, …, *c_l_|l* ≤ ***C***};
7: **for** the *i*-th category, *i* = 1, …, *l*
**do**
8: Define the initial spread value $\sigma_{i}^{\left(0\right)}$ and bandwidth diagonal matrix $\Sigma_{i}^{\left(0\right)}$;
9: Calculate the optimal spread value $\hat{\sigma_{i}}$ of category *c_i_* with a gradient descent algorithm.

$\frac{\partial E}{\partial \sigma_{i}}=\overset{m}{\underset{t=1}{\sum }}\left(\hat{y_{t}}-{\bar{y}}_{t}\right)\frac{\partial \hat{y_{t}}}{\partial \sigma_{i}}\;i\in \left[1,l\right]$


$\hat{\sigma_{i}}=\arg\underset{i\in \left[1,l\right]}{\min}E\left(c_{i}\right)$

10: **end for**
11: Obtain *H* and Σ; training is done.

**Figure 11:**
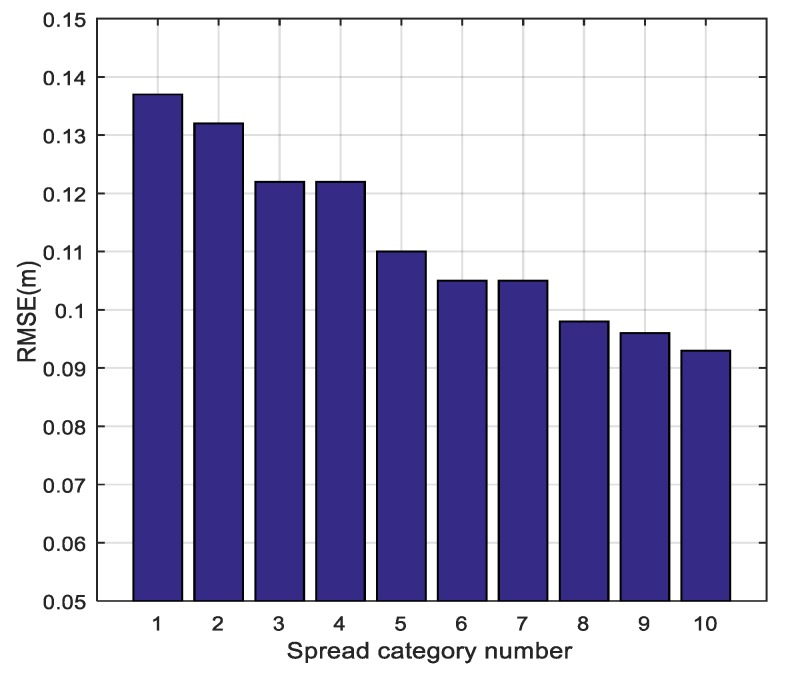
Root-mean-square error (RMSE)of different spread category numbers.

Even if the training result is not optimal, it makes a good compromise between the fitting performance and the algorithm’s complexity. The root-mean-square error (RMSE) results for different spread category numbers are shown in Figure 11. We observe that the fine-grained spread category is a benefit in eliminating the localization error. In our experiment, we defined the category number as 6.

**Figure 12:**
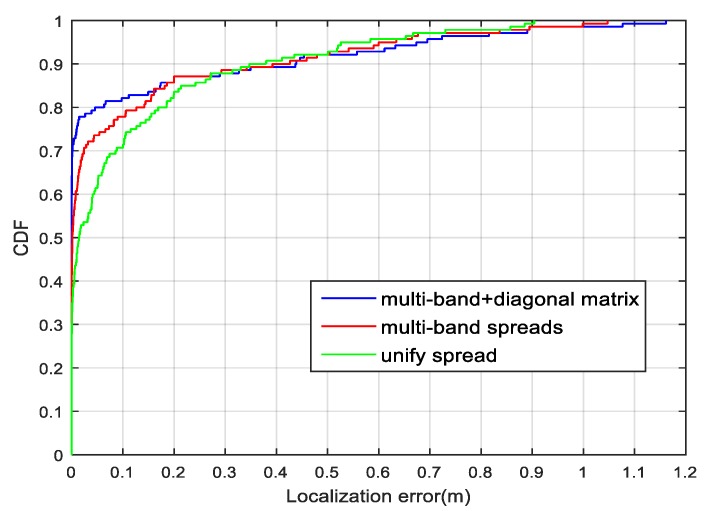
Cumulative distribution functions of localization errors for different spread modes.

We compared the localization performances of the GRNNs trained with different spread strategies, including one unified spread, multiple-bandwidth spreads, and multiple-bandwidth spreads with a diagonal matrix. The localization error is reported as the *L*_2_ norm of the difference between the true position and its estimate. As shown in Figure 12, the multiple-bandwidth spreads and the diagonal matrix can improve the localization accuracy.

#### 4.3.2. Preprocessing Method of Training Data

**Figure 13:**
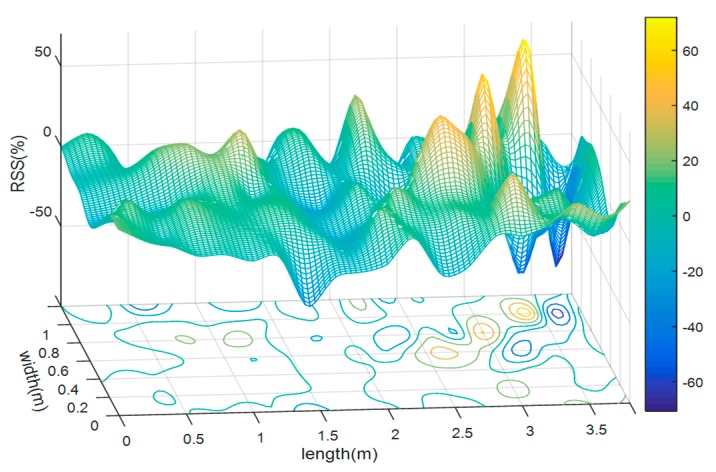
Fluctuation of a random received signal strength (RSS) sample against mean values.

In a dynamic temporal indoor environment, the RSS samples with the same fingerprint suffer from inevitable fluctuations. Thus, the instant RSS value may differ substantially from its mean. As shown by Figure 13, the fluctuation Δ*rss* of a random RSS sample against mean values is obtained following Equation (33).

(33) $$\Delta rss=\frac{RSS_{r}-RSS_{mean}}{RSS_{mean}}\times 100\%$$

where *RSS_mean_* is the mean values calculated by averaging all of the training fingerprint sample sets of the LOS case, and *RSS_r_* is the random value chosen from the evaluation fingerprint sample set.

As every fingerprint datum represents a pattern neuron, a credible training data set is significant to the performance of the GRNN. To reduce the noise and interference in the measured RSS values, the preprocessing method is necessary for fingerprints. Different preprocessing strategies can be employed by the GRNN (including the mean filter and median filter) to refine the RSS fingerprint data. We categorize different kinds of training sample sets from LOS condition as follows:

Set A: all of the collected raw fingerprint samples;
Set B: the mean value set of all of the fingerprint samples;
Set C: the median value set of all of the fingerprint samples;
Set D: the combination of mean and median value sets;
Set E: the mean value set of five fingerprint sample sets.

The results of applying these data sets to train the GRNN and compare the localization accuracies are shown in Table 1. The mean value set is preferred, as the average distance error of the case Set B that is trained is the smallest and requires fewer pattern neurons than in other cases.

#### 4.3.3. Fingerprint Scale of Training Data

**Figure 14:**
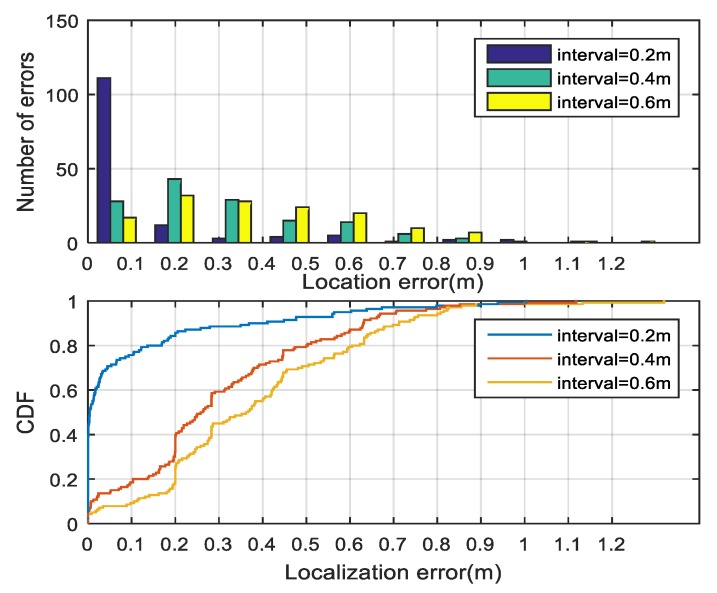
Localization accuracy under different fingerprint scale conditions.

For a fingerprint-based localization system, the scale of the fingerprint data set can directly affect the localization accuracy. Generally, large scale means more fingerprints and better performance at the expense of heavy survey overheads. For a certain area, we can modify the fingerprint scale by tuning the interval between the adjacent reference points. We evaluated the localization accuracy when the node intervals are 0.2 m, 0.4 m, and 0.6 m, respectively. The comparison results are given in Figure 14 and Table 2.

### 4.4. Localization with Outlier Filter

In our experiment, the outlier filtering procedure is illustrated by Algorithm 2. The candidate fingerprints number *k* is 140. The vigilance parameter *ρ* is set as 0.3, the localization algorithm updates the output every 0.1 s, and the target motion velocity was assumed to be less than 2 m/s. We added an extra 0.1 as a safety margin.

**Figure 15:**
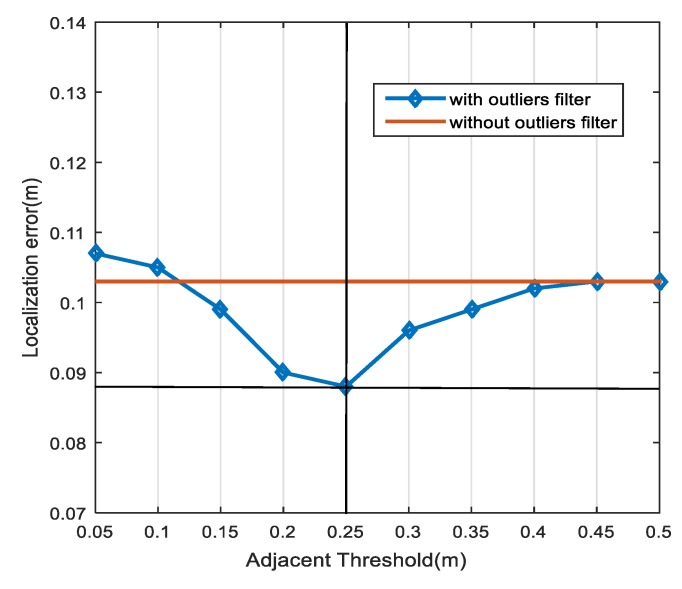
Effect of the adjacent thresholds on the localization accuracy.

The adjacent threshold *v_th_* is the key parameter of the outlier filtering algorithm. We evaluated the localization performance using the tuning parameter *v_th_*. As shown in Figure 15, we observed that when *v_th_* = 0.25 m, the outlier filtering algorithm gains an improved accuracy of 14.6% over the case without it.

**Algorithm 2.** Outlier Filtering 
**Input:**

*k*: number of candidate fingerprints;
*F_c_* = [*c*_1_, *c*_2_, …, *c_k_*]*^T^*: the fingerprint set of candidate nodes;
(*x_i_*, *y_i_*) ∀*i* ∈ [1, *k*]: the location coordinates of *i*-th candidate node;
*v_th_*: the adjacency threshold determined by the cross-validation method;
*ρ*: the upper limit of the distance between two successive estimation outputs.

**Output:**

***Z*** = {*z_i_* | *i* ∈ [0, *k* − 1]}: the outliers index set of candidate notes.
1: Calculate spatial distances between the candidates in *F_c_*, and express the results in matrix *V*
  $V=\left[\begin{array}{ccc} v_{11} & \cdots & v_{1k}\\ \vdots & \ddots & \vdots \\ v_{k1} & \cdots & v_{kk} \end{array}\right]$
2: **if**
*v*_12_ ≤ *v_th_*
**then**
{*c*_1_ is considered as the nearest neighbor of the target;
**goto**;}
3: **else**
{
**for***q* = 1, …, *p*
**do**
  $s_{q}=\{\begin{array}{ll} 1, & \mathrm{if}\;d_{c1,q}\leq d_{c2,q}\\ 0, & \mathrm{otherwise} \end{array},\;\forall q\in \left[1,p\right]$

**end for**

Final score *S* is
  $S=\overset{p}{\underset{q=1}{\sum }}s_{q}-\xi ,\;\left(\xi =\{\begin{array}{cc} 0, & v_{20}>\rho \\ 1, & \mathrm{otherwise} \end{array}\right)$
}
4: Identify outliers in *F_c_*_5_
  $z_{i}=\{\begin{array}{ll} ‘Ture’, & v_{1i}\leq v_{th}\\ ‘Fake’, & \mathrm{otherwise} \end{array}\;\;,\forall i\in \left[2,k\right]$
5: Sent outlier index set *Z* to the GRNN block.

### 4.5. Comparison to Other Methods in LOS Condition

We employed full-scale testing samples for the comprehensive evaluation of and comparison to the positioning performance of the proposed GROF method to that of the original GRNN method, the KNN method, and the BPNN method in the LOS condition. 

In our experiment, the spread value of the GROF was trained into six categories in the diagonal matrix, and the adjacent threshold is defined as 0.25 m. The optimal spread value of the original GRNN was trained by the cross-validation method. In the KNN method, *k* = 4 is based on the lowest RMSE of the validation data. The compared BPNN contains one hidden layer with 80 neurons and uses the hyperbolic tangent activation function. 

The results of the four methods are shown in Table 3. The mean localization error of the GROF is 0.087 m, which is smaller than the 0.103 m of the standard GRNN and the 0.121 m of the BPNN. The performance of the KNN is the worst. The results show that the RMSE performance of the proposed method is up to 15% lower than the traditional GRNN method, up to 29% lower than the BPNN method, and up to 43% lower than the KNN method. 

**Figure 16:**
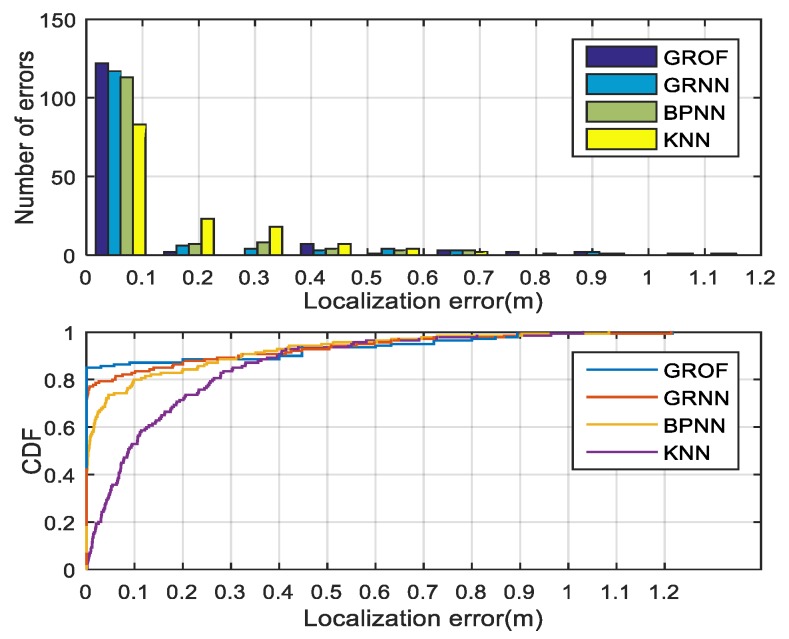
Localization error histogram and cumulative distribution function (CDF) of different algorithm in the line-of-sight (LOS) condition.

The histogram and cumulative distribution function (CDF) of the localization errors for each algorithm are drawn in Figure 16. From the experimental results, we conclude that the localization performance of the proposed GROF method is superior to the KNN algorithm, the standard GRNN algorithm, or the BPNN method. In general, the GROF outperforms the other algorithms in the LOS case.

### 4.6. Comparison to Other Methods in NLOS Condition

Finally, we employed full-scale testing samples for the comprehensive evaluation of and comparison to the positioning performance of the proposed GROF method to that of the original GRNN method, the KNN method, and the BPNN method in the NLOS condition.

In the first case, the object area was firstly divided into two subareas, as shown in Figure 10a. The spread values of the GROF were trained into five categories in the diagonal matrix, and the adjacent threshold is defined as 0.25 m in each subarea. The optimal spread values of the original GRNN were trained by the cross-validation method. In the KNN method, *k* = 4 based on the lowest RMSE of the validation data. The compared BPNN contains one hidden layer with 80 neurons and uses the hyperbolic tangent activation function.

In the second case, the object area was also divided into two subareas by the wall. The spread values of the GROF were trained into six categories in the diagonal matrix for both subareas, and the adjacent thresholds were defined as 0.25 m. The other experimental parameters are the same as the values in Case 1.

In the third case, the object area was divided into three subareas, as shown in Figure 10c. The spread values of the GROF were trained into five categories in the diagonal matrix for the two room subareas, while four categories for the corridor subarea. The other experimental parameters are the same as in the former cases.

**Figure 17:**
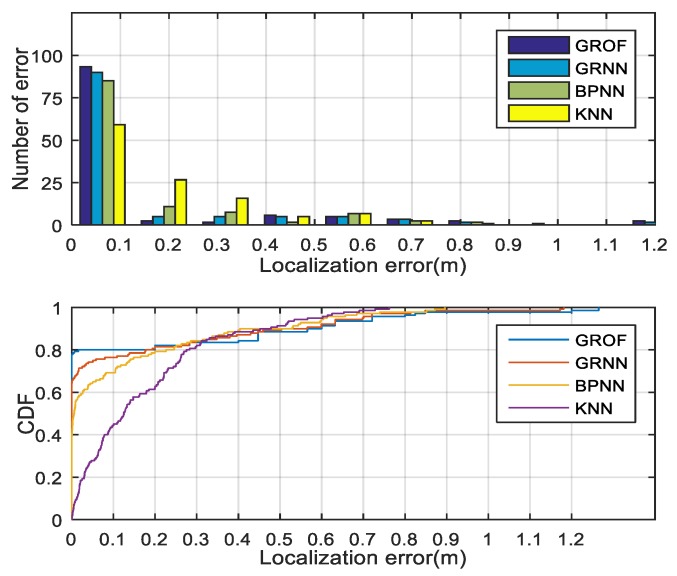
Localization error histogram and CDF of different algorithm in NLOS Case 1.

**Figure 18:**
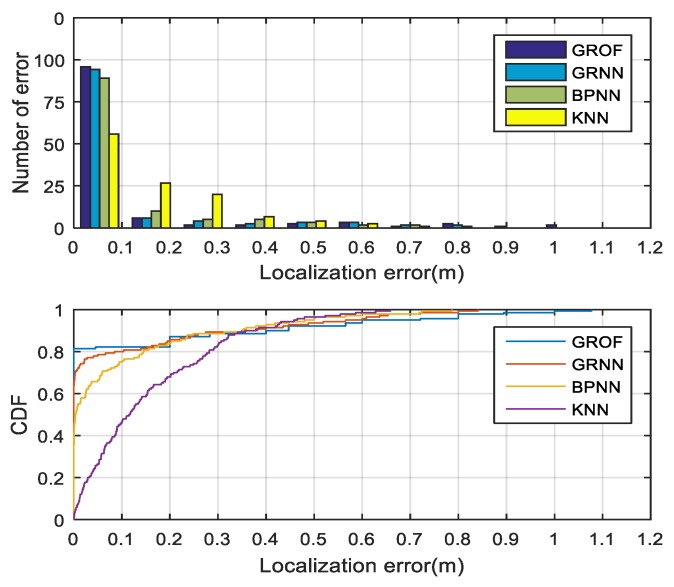
Localization error histogram and CDF of different algorithm in NLOS Case 2.

**Figure 19:**
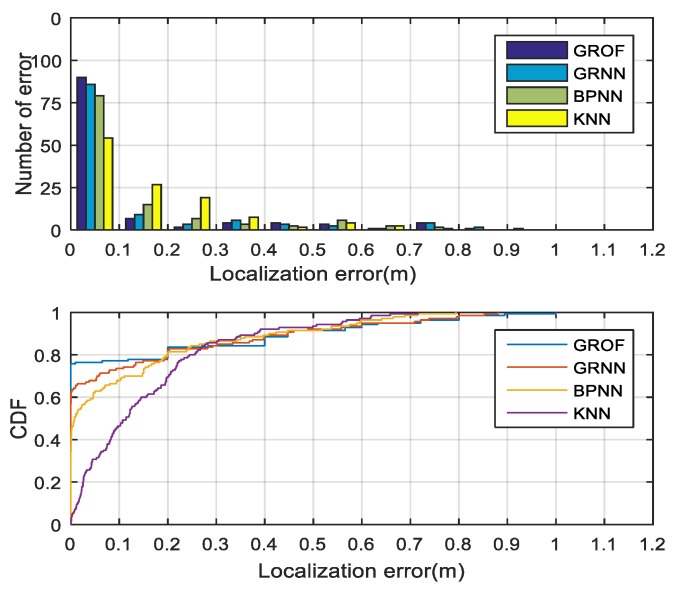
Localization error histogram and CDF of different algorithm in NLOS Case 3.

The histogram and cumulative distribution function (CDF) of the localization errors for each algorithm in three NLOS cases are drawn in Figure 17, Figure 18 and Figure 19. It can be seen that, although the RMSE performance of the proposed method is better than the other algorithms, it was not ideal to control the maximum error while the KNN algorithm achieved lower maximum error. The main reason for this result was that, because of the partly over-fitting of GROF algorithm, the error of some estimates became larger than the other algorithms, and the fitting performance of the GROF algorithm can be improved as the number of training samples increases. As in the LOS case, we used five times more training samples, the maximum error performance of the proposed method was similar to other algorithms’. Moreover, in the actual positioning scenario, some of the large estimation errors could be reduced by combining some constraint methods, such as the Kalman filtering algorithm.

**Figure 20:**
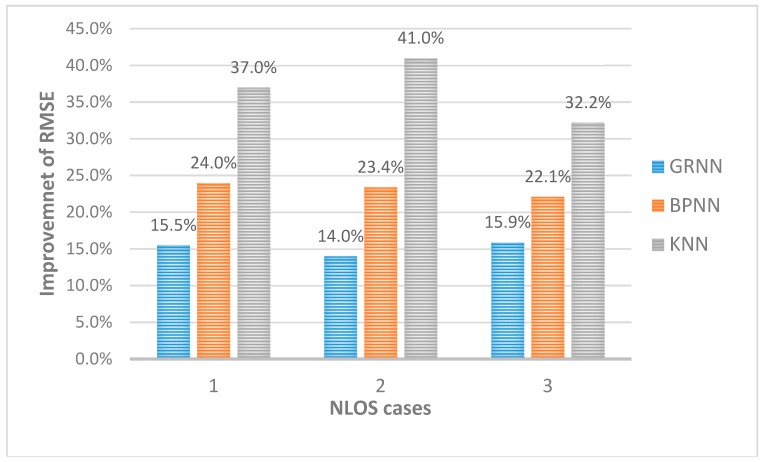
The RMSE improvement of GROF versus different algorithms in three NLOS cases.

The results of the four methods in different NLOS conditions are shown in Table 4, Table 5 and Table 6. Compared to LOS condition, the localization performance of every algorithm degrades. In NLOS Case 1, the mean localization error of the GROF is 0.109 m, which is smaller than the 0.129 m of the standard GRNN and the 0.144 m of the BPNN. The performance of the KNN is the worst. The results show that the RMSE performance of the proposed method is up to 15.5% lower than the traditional GRNN method, up to 24% lower than the BPNN method, and up to 37% lower than the KNN method. In NLOS Case 2 and Case 3, the results are similar to Case 1, where the GROF outperforms the other algorithms, as shown in Figure 20.

According to the above experimental results, we conclude that the localization performance of the proposed GROF method is superior to the KNN algorithm, the standard GRNN algorithm, or the BPNN method. In general, the GROF outperforms the other algorithms in the NLOS condition.

## 5. Conclusions

In this work, a novel indoor positioning approach, GROF, is proposed to promote the positioning accuracy and robustness. By adapting to the characteristics of indoor positioning, we adopt a new kind of multiple-bandwidth kernel architecture to achieve a more flexible regression performance than the traditional GRNN, without the extra training sample requirement. The proposed multiple-bandwidth kernel spread training method leverages the geographical correlation of the fingerprints, that the adjacent kernels are supposed to be neighbors in the ground truth. It is flexible and significant that the tunable spread scale is beneficial to achieve a good tradeoff between the performance and complexity. Furthermore, when the object area is very large or in a NLOS condition, the proposed method still works by dividing the area into several small and fingerprints-convex areas. Then, the spread training process runs in each subarea separately and generates a part of the neurons in the pattern layer of the GRNN. As long as it is assembling the trained pattern neurons from all of the subareas, the spread training procedure in the NLOS condition is successfully achieved. In addition, an outlier filter scheme method is embedded into the localization module, to alleviate the impacts of environmental changes. The experimental results show that the proposed GROF method outperforms the positioning methods based on the standard GRNN, KNN, or BPNN methods, in localization accuracy both in the LOS and NLOS conditions.

In this paper, our primary objective is to develop the localization method for static signal source. During the survey process, fingerprint data were measured in a dynamic indoor environment with random people movement; furthermore, we also considered the movement velocity factor in the outlier filter algorithm.

## Figures and Tables

**Table 1:** Performance comparison of different training data sets.

Training Data	Pattern Quantity	RMSE (m)
Set A	140 × 80	0.21
Set B	140	0.1
Set C	140	0.12
Set D	140 × 2	0.1
Set E	140	0.14

**Table 2:** Performance comparison of different fingerprint scales.

RP Interval (m)	Fingerprints Quantity	RMSE (m)
0.2	140	0.1
0.4	40	0.31
0.6	21	0.39

**Table 3:** Accuracy and precision of different algorithms in the line-of-sight (LOS) condition.

Method	RMSE (m)	RMSE < 0.1 m	RMSE < 0.2 m
GROF	0.087	88.9%	90.2%
GRNN	0.103	82.6%	87.4%
BPNN	0.121	78.1%	82.8%
KNN	0.152	55%	72.1%

**Table 4:** Accuracy and precision of different algorithms in NLOS Case 1.

Method	RMSE (m)	RMSE < 0.1 m	RMSE < 0.2 m
GROF	0.109	80.9%	82.1%
GRNN	0.129	75.6%	80.2%
BPNN	0.144	69.2%	77.8%
KNN	0.173	47.6%	63.5%

**Table 5:** Accuracy and precision of different algorithms in NLOS Case 2.

Method	RMSE (m)	RMSE < 0.1 m	RMSE < 0.2 m
GROF	0.098	83.3%	86.0%
GRNN	0.114	79.9%	84.2%
BPNN	0.128	74.3%	82.5%
KNN	0.166	48.7%	68.4%

**Table 6:** Accuracy and precision of different algorithms in NLOS Case 3.

Method	RMSE (m)	RMSE < 0.1 m	RMSE < 0.2 m
GROF	0.116	78.2%	80.2%
GRNN	0.138	74.0%	79.5%
BPNN	0.149	68.4%	78.0%
KNN	0.171	48.6%	64.5%

## Appendix A

Assuming there are *n* fingerprint RPs uniformly distributed in a rectangle area; given that *a* × *b* = *n*, *a* and *b* are the number of points on the horizon side and the vertical side, respectively. Then, we can easily obtain the calculation amount, *H*, of the proposed algorithm, according to Equation (A1)

(A1) $$\begin{array}{ll} H & =a(b-1)+b(a-1)\\ & =2ab-a-b\\ & =2n-(a+\frac{n}{a}) \end{array}$$

Apparently, when $a=b=\sqrt{n}$, the maximum of $H=2\left(n-\sqrt{n}\right)$ is obtained.

As the computational overhead of the traditional approach is G=n(n−1)/2, the proposed algorithm can reduce the calculation amount using Equation (A2)

(A2) $$\begin{array}{ll} \frac{G}{H} & =\frac{n(n-1)}{4(n-\sqrt{n})}\\ & =\frac{\left(n-\sqrt{n})(n+\sqrt{n}\right)}{4(n-\sqrt{n})}\\ & =\frac{\left(n+\sqrt{n}\right)}{4} \end{array}$$

When the environment is not rectangular or it contains multiple rectangular sub-areas, it can be assumed that there are *n* fingerprint RPs distributed in two rectangular subareas with *n*_1_ and *n*_2_ RPs, respectively. The maximum overall calculation amount, *H_all_*, can be obtained according to Equation (A1); and it is easy to get the result that *H_all_* is smaller than *H* from (A3).

(A3) $$H_{all}=2\left(n_{1}-\sqrt{n_{1}}\right)+2\left(n_{2}-\sqrt{n_{2}}\right)=2\left[\left(n-(\sqrt{n_{1}}+\sqrt{n_{2}}\right)\right]<2\left(n-\sqrt{n}\right)\;$$

As the computational overhead of the traditional approach is still *G* = *n*(*n* − 1)/2, the calculation amount could be reduced even further by the proposed algorithm, compared to one rectangular area case.
